# First steps of learning analytics in a blended learning general practice curriculum at Saarland University – a quantitative approach

**DOI:** 10.3205/zma001653

**Published:** 2023-11-15

**Authors:** Helene Junge, Kerstin Schuster, Aline Salzmann, Sara Volz-Willems, Johannes Jäger, Fabian Dupont

**Affiliations:** 1 Saarland University, Department of Family Medicine, Homburg, Germany

**Keywords:** formative assessment, digital learning behaviour, blended learning, undergraduate medical education

## Abstract

**Objectives:**

Medical education has been revolutionized by the growing importance of digital learning. Little is known about students’ online study behaviour and its relationship with exam performance. This quantitative study analyses and describes students’ digital learning behaviours in a blended learning curriculum for General practice at Saarland University, Germany. It also examines the relationship between digital learning behaviour and exam performance.

**Methods:**

Cohort and individualized AMBOSS^®^ user data from 195 students at Saarland University was analysed quantitatively. Performance in course-specific multiple-choice question sessions and user data of the integrated online learning activities were correlated with each other and with General practice exam grades. Anonymized data from 10,534 students from 35 other German universities served as the reference cohort. Differences in digital learning behaviour between the groups were calculated using Mann-Whitney-U-Test for non-normally distributed data.

**Results:**

Students in the blended learning course used integrated content more frequently than the reference cohort (U=48777, p<0.001). The number of digital learning cards read correlated moderately with digital formative assessment performance (ρ=0.331, p=0.005 and ρ=0.217, p=0.034). Formative assessment scores and exam results correlated strongly in the summer semester cohort (ρ=0.505, p<0.001), and moderately in the winter semester cohort (ρ=0.381, p<0.001).

**Conclusion:**

There is a difference in the usage of online learning activities when they are purposefully integrated into a curriculum. Digital learning activities including formative assessment may serve as valuable, constructively aligned exam preparation. This is relevant for medical educators when planning future blended learning curricula and portfolio systems, as it may save financial and human resources.

## Zusammenfassung

**Hintergrund:**

Die medizinische Ausbildung wurde durch die wachsende Bedeutung des digitalen Lernens revolutioniert. Über das Online-Lernverhalten der Studierenden und dessen Zusammenhang mit den Studienleistungen ist wenig bekannt. Diese quantitative Studie analysiert und beschreibt das digitale Lernverhalten der Studierenden in einem Blended-Learning-Curriculum für Allgemeinmedizin an der Universität des Saarlandes, Deutschland. Daneben wird der Zusammenhang zwischen digitalem Lernverhalten und Prüfungsleistungen untersucht.

**Methoden:**

Kohorten- und individualisierte AMBOSS^®^ Nutzerdaten von 195 Studierenden der Universität des Saarlandes wurden quantitativ ausgewertet. Die Leistungen in den kursspezifischen Multiple-Choice-Fragesitzungen und die Nutzerdaten der integrierten Online-Lernaktivitäten wurden miteinander und mit den Noten der Klausur im Fach Allgemeinmedizin korreliert. Als Referenzkohorte dienten anonymisierte Daten von 10.534 Studierenden von 35 anderen deutschen Universitäten. Unterschiede im digitalen Lernverhalten zwischen den Gruppen wurden mit dem Mann-Whitney-U-Test für nicht-normalverteilte Daten berechnet.

**Ergebnisse:**

Die Studierenden des Blended-Learning-Kurses nutzten integrierte Inhalte häufiger als die Referenzkohorte (U=48777, p<0,001). Die Anzahl der gelesenen digitalen Lernkarten korrelierte moderat mit der Leistung im formativen Assessment (ρ=0,331, p=0,005 und ρ=0,217, p=0,034). Die Ergebnisse des formativen Assessments und die Prüfungsergebnisse korrelierten in der Kohorte des Sommersemesters stark (ρ=0,505, p<0,001) und in der Kohorte des Wintersemesters moderat (ρ=0,381, p<0,001).

**Schlussfolgerungen:**

Es gibt einen Unterschied in der Nutzung von Online-Lernaktivitäten, wenn diese gezielt in einen Lehrplan integriert werden. Digitale Lernaktivitäten, die formatives Assessment enthalten, können als wertvolle, konstruktiv ausgerichtete Prüfungsvorbereitung dienen. Dies ist für Lehrende in der Allgemeinmedizin bei der Planung künftiger Blended-Learning-Lehrpläne und Portfoliosysteme von Bedeutung, da so finanzielle und personelle Ressourcen eingespart werden können.

## 1. Introduction

In recent years, online learning has reshaped the way undergraduate medical students study for the subject of General practice (GP). With the development of new content such as apps, podcasts, question banks and online learning platforms, online learning activities (OLA) have become essential to a modern learning environment [1], [2]. These tools will likely be vital to the training of upcoming physicians, especially since the global COVID-19 pandemic has further accentuated remote learning [3], [4], [5]. Previous studies have suggested high acceptance of online learning among students [6]. There is also evidence that blended learning (BL) may be superior to traditional onsite curricula regarding knowledge outcome [7], [8]. At the same time, there appears to be a lack of institutional support and limited guidance on how online learning may be implemented in at medical schools [9], [10], [11], [12]. 

A recently emerging practice to track and store students’ web-based learning behaviour and make it evaluable for teachers in health sciences is the application of so-called “learning analytics” [13]. Although a great amount of learning data is already collected through digital learning platforms in medical education, the practice of “learning analytics” is still relatively new to medical schools, and has rarely been used for GP undergraduate training [14]. 

Learning analytics can consider various data resources, e.g., number of log-ins to e-learning platforms or time spent on e-learning platforms [13]. Another tool that can be used for “learning analytics” is formative assessment (FA). Previous studies have shown that FA positively impacts learning and academic performance by providing students with feedback and guidance on their learning process [15], [16], [17]. Although traditionally associated with summative assessment, multiple choice questions (MCQ) can equally be used for FA in medical education and are popular among learners [15], [16], [17], [18], [19]. Previous studies show that repeated testing throughout the learning process may enhance long-term retention of information in students [20], [21]. The “testing effect” describes the circumstance that if one or more “tests” are included in exam preparation, students show better exam performance, even if the exam consists of novel, more challenging questions [21]. For MCQ to serve as a FA tool, a hint and/or results explanation should be provided upon answering each question, since feedback is an essential component of FA [22], [23]. It has also been stated that FA works best if built into a planned curriculum [22]. Based on these findings, MCQ may be a valuable tool for FA exercises in BL curricula, which again can be used as a tool for learning analytics.

At Saarland University (UdS), various OLA such as podcasts, MCQ and learning cards are incorporated into a new compulsory year 5 GP BL curriculum. Some OLA used in the course, namely MCQs and learning cards, are provided by AMBOSS^®^, a common provider of OLA in undergraduate medical education in Europe and the US [24]. As previous studies have shown correlations between digital learning behaviour and exam performance, this study aims to investigate this relationship for a BL course in undergraduate GP [25], [26]. By analysing students’ use of digital learning cards and FA sessions during the GP course, and correlating them with GP exam performance and first state exam grades, this quantitative study aims to answer the following questions: Is there is a correlation between learning card usage and FA performance? Is there a correlation between FA performance and GP exam performance? By comparing user data from two different semester cohorts at UdS and a nationwide reference cohort, this study also aims to examine: Is there a difference in the use of digital learning resources at Saarland University between students in the summer and winter semester? How do UdS students use digital learning cards that are integrated in a GP BL curriculum in comparison to a nationwide cohort?

## 2. Methods

### 2.1. Participants and setting

Participants were year-5 medical students at UdS who participated in the compulsory GP course during winter semester 2020/21 or summer semester 2021. Inclusion criteria were consent to participate via an online form, an active AMBOSS^®^ account, provision of the registration email address and participation in the 60-item final examination for the GP BL course. Usage fees for AMBOSS^®^ were covered by the university for all students regardless of participation. 86 (93%) winter semester students and 109 (98%) summer semester students consented to study participation. 34 learning cards and nine especially designed question sessions with 30 questions each (“UdS sessions”) were selected from the AMBOSS^®^ database for the BL course. Selected content was integrated into customized online material for GP, such as commentaries, podcasts, screen- and video casts and online lectures, on the curriculum’s homepage. Students were able to access the homepage throughout the semester. While they were able to complete the MCQ sessions at any given time, it was recommended to do so after studying the associated material. To avoid learning effects from repeated completion, the score of each first MCQ session attempt was used for data analysis. Correct MCQ results with explanations were provided upon answering.

### 2.2. Data collection

AMBOSS^®^ user data of participating UdS students was recorded for the winter semester (01/10/20-28/02/21) and summer semester (01/04/21-31/08/21). The dataset contained user data from the UdS FA sessions, namely the number of questions answered, and the rate of questions answered correctly on first attempt (“FA score”). It also included the rate of all questions answered correctly on AMBOSS^®^ (“question success rate”). Additionally, the number of learning cards read and the number of accesses for the learning cards were recorded, both for the “selected learning cards” for the BL course and for all learning cards on AMBOSS^®^. To complete the information obtained through the AMBOSS^®^ data set, UdS students were asked to provide information about their socio-demographic data and grade in the completed first state examination. 10,393 anonymized AMBOSS^®^ users from 35 different German universities served as a reference cohort. Reference cohort data was collected between 01/10/2020 and 28/02/21. Users were included if they were in their fifth year of medical school, had been provided access to AMBOSS^®^ by their university free of charge, and had accessed at least one OLA on AMBOSS^®^ during the respective timeframe.

### 2.3. Data analysis

Analyses were performed using Jamovi (Version 1.6.23.0). Descriptive analyses included mean, median and standard deviation. To investigate the relationship between learning card use, FA performance, state exam grades and exam performance, correlation analysis was conducted for both semester cohorts separately. Differences in data between summer and winter semester cohort and between UdS students and reference cohort were calculated with Mann-Whitney-U-Test (U). All analysed data were non-normally distributed (Shapiro-Wilk-Test). For all analyses, an alpha significance level of 0.05 with a two-sided approach was used. Power analysis was done for a two-sided t test prior to data collection with G*power and jpower (Jamovi). Effect sizes (correlation coefficients) were evaluated using Spearman’s rho (ρ) for non-normally distributed data (ρ<0.3: small effect; ρ=0.3-0.5; moderate effect, ρ>0.5; large effect). Correlation coefficients were also freely interpreted based on the same effect size levels, to maintain readability [27]. Benchmarks for effect sizes were applied as suggested by Cohen [28], [29]. For state exams, the German grading system (1=A to 6=F) was used. 

## 3. Results

### 3.1. Sociodemographic data

Mean age of participants was 26.3 (SD: 4.61) years in the winter semester, and 24.2 years (SD: 2.35) years in the summer semester. Among participants in the winter semester, 52 (60.5%) of participants were female and 34 (39.5%) were male. In summer semester the distribution was 63 (58.3%) and 45 (41.7%) respectively. One student in the summer semester did not provide their gender.

### 3.2. Digital learning behaviour

Digital learning behaviour data was available for 98 (90%) summer semester students and 73 (85%) winter semester students. There was no significant difference between the mean number of selected learning cards read between summer- and winter semester students (31±4.7 vs. 30±5.0; U=3046, p=0.092). At the same time, the 34 selected learning cards were accessed significantly more often by summer semester students than by winter semester students (185±143 vs. 131±71.7; U=2726, p=0.008). There was a strong correlation between the number of selected learning cards read and the frequency of accesses for the selected learning cards, both in the summer semester (ρ=0.525, p<0.001, see table 1 (Tab. 1)) and the winter semester (ρ=0.632, p<0.001, see table 2 (Tab. 2)). Again, summer semester students read significantly more learning cards, considering all learning cards on AMBOSS^®^, than winter semester students (665±348 vs. 313±240; U=1561; p=0.001). Students from the reference cohort who did not take part in the BL curriculum read significantly fewer of the selected learning cards, on average only 10 out of the 34 cards (SD: 8.28; U=48200, p<0.001). On average, the reference cohort also accessed the selected learning cards significantly less frequently than the UdS cohort (38±54.2 vs. 162±120; U=157892; p=0.001).

### 3.3. Digital learning behaviour and formative assessment 

Weak and moderate correlations were found between the number of selected learning cards read and the FA score (winter semester: ρ=0.331, p=0.005; summer semester: ρ=0.217, p=0.034, see table 1 (Tab. 1) and table 2 (Tab. 2)). A weak correlation was found between the number of accesses for selected learning cards and the FA score in both semesters (winter semester: ρ=0.275, p=0.02; summer semester: ρ=0.281, p=0.005, see table 1 (Tab. 1) and table 2 (Tab. 2)).

### 3.4. Formative assessment performance

User data for the UdS MCQ sessions was available for 106 (97%) summer semester students and 80 (93%) winter semester students. On average, out of 180 MCQ selected for the BL course, UdS students answered 92% (166, SD: 33.6) in the summer semester and 96% (172, SD: 23.1) in the winter semester. The mean result score for the initial session (“FA score”) was 0.860 (SD: 0.0841) for summer semester students, hence significantly higher than the score for winter semester students (0.789, SD: 0.112, U=2452, p=<0.001). In total, summer semester students answered significantly more questions on AMBOSS^®^ than winter semester students (6496±4574 vs. 1276±1977; U=856, p<0,001).

In both UdS semester cohorts, the overall question success rate on AMBOSS^®^ was correlated positively with FA scores. This correlation was strong for the summer semester cohort (ρ=0.789, p<0.001, see table 1 (Tab. 1)) and moderate for the winter semester cohort (ρ=0.419, p<0.001, see table 2 (Tab. 2)). 

### 3.5. Formative assessment performance and exam performance

85 (99%) winter semester and 107 (98%) summer semester students participated in GP final exam. There was a strong correlation between FA scores and GP exam results in the summer semester cohort (ρ=0.505, p<0.001, see table 1 (Tab. 1)), and a moderate correlation in the winter semester cohort (ρ=0.381, p<0.001, see table 2 (Tab. 2)). Correspondingly, moderate correlations between scores in the first state exam and FA scores were found in both the summer semester cohort (ρ=0.332, p<0.001, see table 1 (Tab. 1)) and the winter semester cohort (ρ=0.414, p<0.001, see table 2 (Tab. 2)).

A strong correlation was found between the overall question success rate and the exam scores of UdS students in the summer semester (ρ=0,568, p<0.001). No significant correlation was found for the winter semester cohort. 

## 4. Discussion

### 4.1. Summary 

This study explores interrelations between students’ use of OLA, FA performance, and exam performance for a GP curriculum. Results indicate that embedding online learning content into a BL curriculum increases its use. Intensity of digital GP content use is related to scores in constructively aligned FA exercises. Scores in FA exercises appear to correlate with exam performance. 

### 4.2. Summer vs. winter semester cohort

Results show distinct differences in online learning behaviour between the summer and winter semester cohort. Students at UdS usually prepare for the state examinations during the summer semester. This may explain greater use of OLA and stronger performance in FA sessions for summer semester students. Due to the greater use of MCQs as a tool for state exam preparation, summer semester students may have been more familiar with FA as an OLA. This could explain the fact that a strong correlation between the overall question success rate on AMBOSS^®^ and exam scores was found for the summer semester-, but not for the winter semester cohort. These results may indicate that FA is particularly useful for assessing exam performance when used frequently and intensively. It may also suggest that the motivation to study and the acceptance of OLA and FA appear to be higher when aligned with state exam content, especially in the semester prior to the state exam.

### 4.3. Digital learning and digital formative assessment 

Findings show that it is possible to promote students’ online learning efforts in GP by pre-selecting OLA and then specifically incorporating them into a curriculum. In the UdS cohort, a higher amount of selected learning cards was read compared to the nation-wide reference cohort. These learning cards were also accessed more frequently, indicating that the integration of OLA in the course resulted in greater traffic for the GP learning cards. The strong correlation between the number of selected learning cards read overall and the frequency of accesses of those cards may indicate that students who read more learning cards for the BL course also studied them more frequently. This may indicate that the integration of OLA into a curriculum incentivises its use by students. 

An interesting finding of this study is that FA scores could be used to monitor exam performance during a GP course, since FA scores correlate strongly with GP subject exam scores and moderately with scores for the first state exam. This could help medical educators quickly identify and support weaker learners in a course, even before summative assessment takes place. In the future, these findings may help reduce the focus on summative assessment as sole proof of performance in GP medical education.

### 4.4. Implications for practice

Literature shows an extensive use of OLA for self-directed learning and high popularity of self-monitoring via FA among medical students [6], [30]. Our study adds to previous research that describes positive correlations between students’ results in self-assessment exercises and subject examinations in other countries, for other medical subjects [16], [31], [32], [33]. Since students tend to choose learning interfaces they are familiar with, integrating established OLA into curricula may help promote online learning even further [34]. 

To date, online learning content and online FA exercises have mostly been developed specifically for university-specific blended learning courses [35], [36], [37], [38]. As stated by Prober et al. in 2013, it can cause frustration and stress among students when course curricula do not mirror the content of standardized national examinations [39]. That may be a reason why students often prefer third-party study material over faculty-specific course content [39]. Using an existing online learning tool that is adapted to the local national examination and already well known among students could save faculty time and staff resources. Unlike with faculty-specific formative assessment systems, little additional programming and design effort is required when using an existing learning platform. This is especially relevant for a subject like GP, where often teaching staff are doctors working in practices simultaneously, and university institutes tend to be small. Based on existing literature and our findings, it may be useful to purposefully integrate third-party OLA, including FA into BL courses to increase the effectiveness of digital learning. As the example of the GP BL course at UdS shows, they may be a useful complement to faculty-specific online or in-person content. Evidently, medical school curricula first and foremost serve the purpose of training good doctors, not just preparing students for state exams. Nevertheless, constructive alignment of course content and state examination requirements may foster learner motivation and learning success since “assessment drives learning”. This is especially true since the national state examinations are often the main common denominator among different medical schools [39]. 

It has been stated that the use of MCQ for FA is limited by the one-dimensionality of this assessment format. MCQ may enable a learner to correctly answer a question by solely recognizing the correct answer (“cueing”) [40]. For this reason, the use of key-feature MCQ to evaluate clinical reasoning is currently being investigated at UdS. In key-feature question sequences, going back to a previous question is not possible. This way, multistage decision processes can be simulated, even if additional information is given in subsequent items [41]. Key-feature questions may also reduce cueing [40]. Research supports the use of key-feature questions to assess clinical reasoning [42]. Besides investigating further strategies to improve the MCQ format, future studies should investigate the correlations found in this study for other forms of evaluation, such as OSCEs.

For medical educators in GP, collecting and analysing students’ online learning behaviour data in the sense of learning analytics may enable continuous performance tracking of both individual students and cohorts, especially during times of remote learning [43], [44], [45]. Lockyer et al. (2017) stated that digital tools that facilitate the collection and analysis of assessment data will be crucial for future competency-based medical education, for example by feeding into e-portfolios [46]. E-portfolios provide a longitudinal view on learning and enable teachers to monitor students’ learning success while the course is still in progress. Especially when financial or humanitarian resources are lacking, automated assessment and feedback, e.g., through learning analytics and E-portfolios, can provide relief. In the future, E-portfolios that include FA could even provide an alternative to summative assessment [46], [47], [48], [49], [50], [51].

### 4.5. Limitations

Due to incorrect email addresses, AMBOSS^®^ user data of four students from the winter semester and one student from the summer semester could not be matched with consent forms. This data was not considered for analysis. 

Although only fifth-year students were considered for the reference cohort, the curricula of the various German medical schools differ. It remains unclear which subjects the students of the reference cohort were taught in their fifth year and whether and to what extent digital learning was included in other curricula.

As part of an ongoing cooperation with IMPP, both FA exercises and the GP exam at UdS are based on state exam questions. It was ensured that no GP exam questions were used in the FA sessions. However, it is evident that the use of MCQs for FA is limited since MCQ can only assess factual knowledge acquired in a course. The BL course at UdS, like many other BL courses, is designed to teach not only cognitive, but also affective competencies such as empathy and communication skills, which are trained in on-site simulations. These abilities are especially important for the subject of GP and cannot be measured well with MCQs. In the future, other types of assessment, e.g., OSCEs, need to be adapted for a digital learning environment and included in e-portfolios for GP. 

## 5. Conclusion

This study provides some evidence that the selection and embedding of OLA in a digital GP learning environment can increase their use. Digital, self-directed FA may help predict students’ exam performance. Learning analytics of students’ online learning behaviour can promote learning success in GP, while helping educators to guide and monitor students’ self-directed learning. This is especially relevant during periods of online learning and when human resources are scarce. In the future, learning analytics may be a useful part of e-portfolios and even help replace or transform traditional forms of summative assessment.

## Abbreviations

- BL: blended learning
- FA: Formative Assessment
- FA Score: Formatives Assessment Score
- GP: General practice
- IMPP: Institut für medizinische und pharmazeutische Prüfungsfragen
- MCQ: Multiple choice questions
- OLA: Online learning activities
- UdS: Universität des Saarlandes
- SD: Standard deviation
- Vs.: Versus

## Ethics approval

Ethics approval was obtained prior study initiation by Saarland medical association ethics committee on 25.09.2020 (Bu234/20).

## Competing interests

The authors declare that they received financial and structural support by AMBOSS^®^, the Medical Faculty of Saarland University and the Kassena¨rztliche Vereinigung Saarland. The department for GP Homburg has cooperation agreements with AMBOSS^®^ and the IMPP as external parties. No external party had any influence on study design, data collection, analysis, or publication procedures.

## Figures and Tables

**Table 1:**
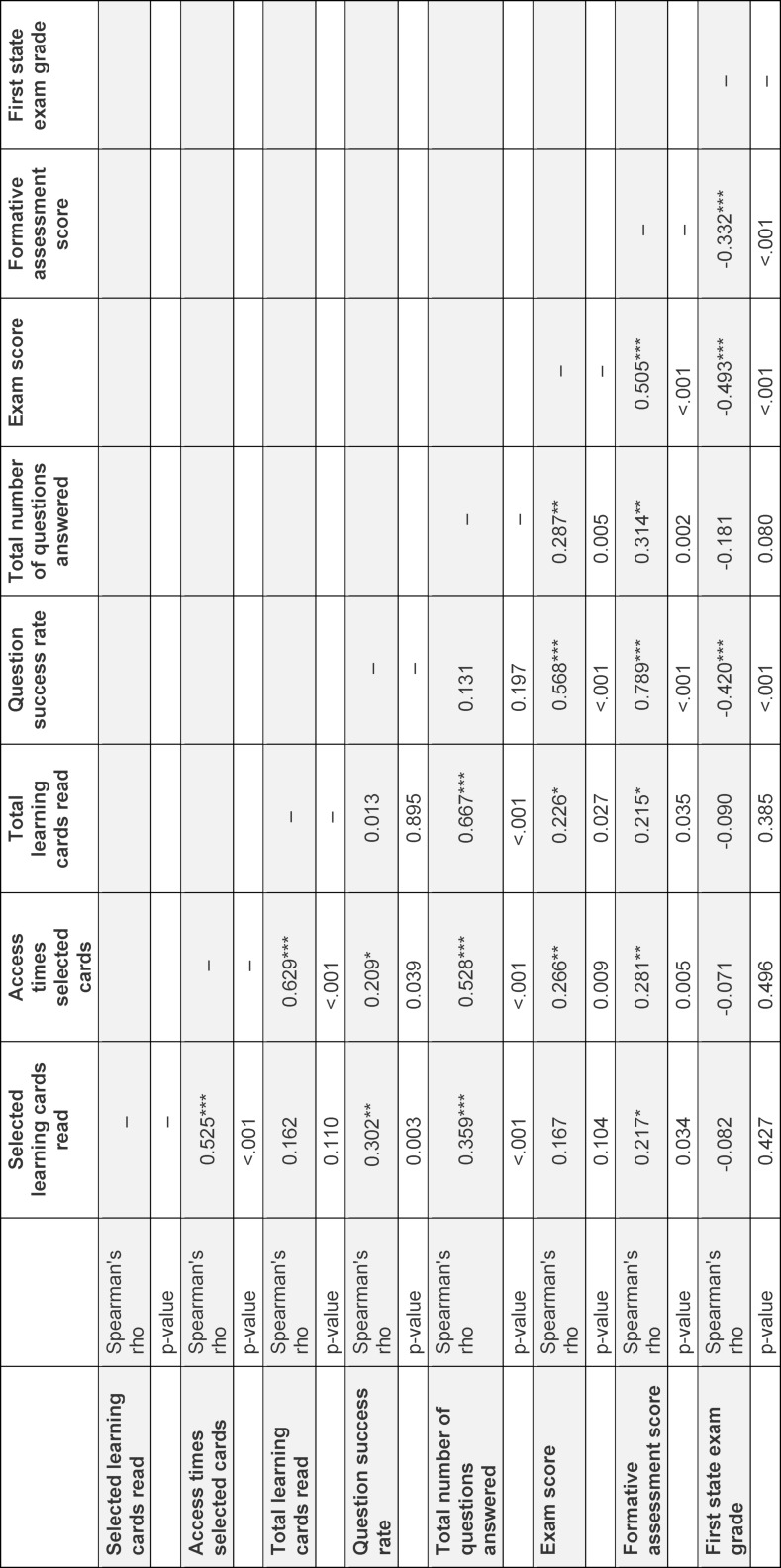
Online learning behaviour, formative assessment scores and exam performance for Students at Saarland University in the summer semester 2021 (correlation table).

**Table 2:**
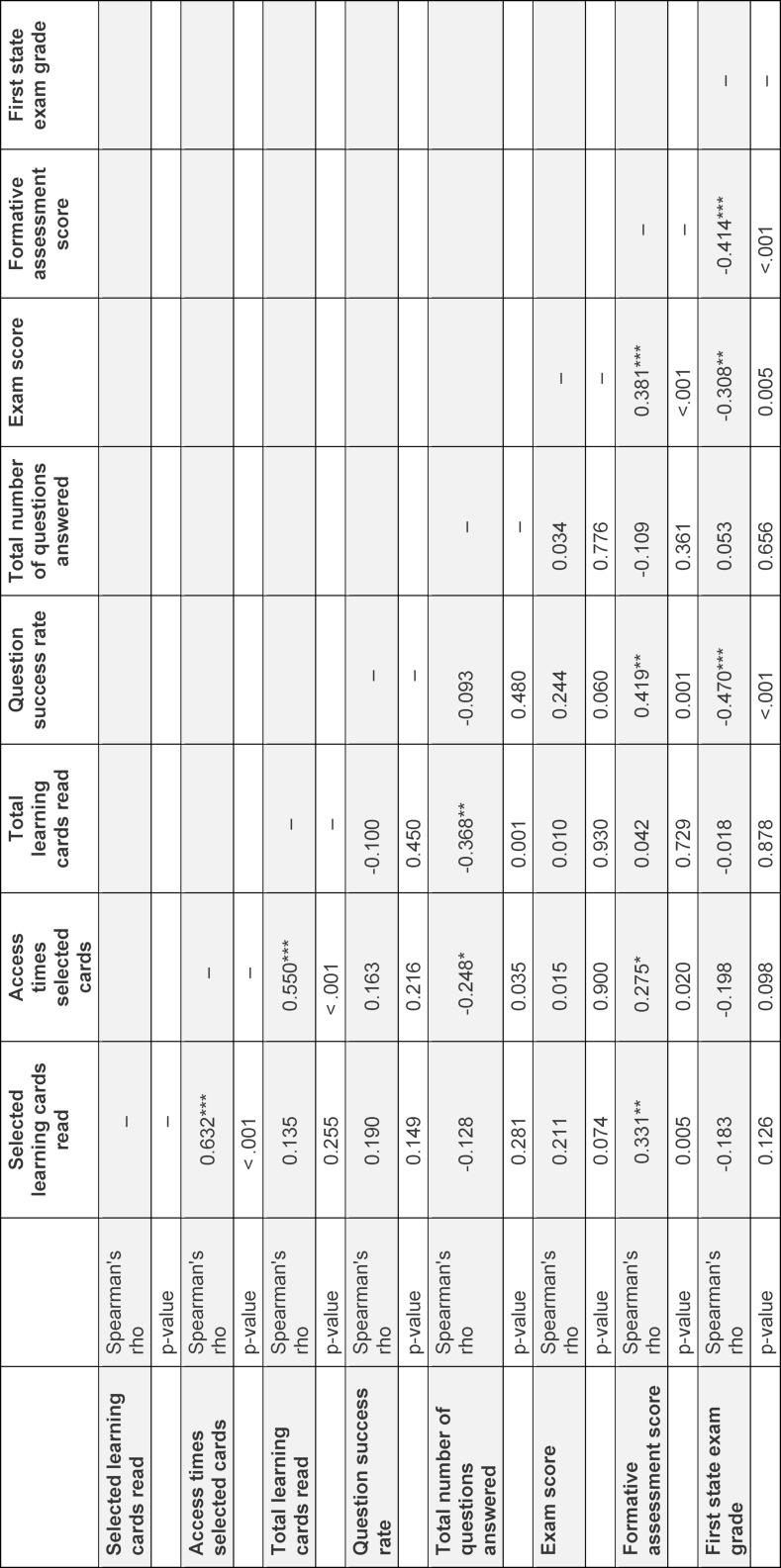
Online learning behaviour, formative assessment scores and exam performance for Students at Saarland University in the winter semester 2020/21 (correlation table).
